# Simultaneous Quantitation of Clevidipine and Its Active Metabolite H152/81 in Human Whole Blood by LC-MS/MS: Application to Bioequivalence Study

**DOI:** 10.3389/fchem.2022.861952

**Published:** 2022-04-07

**Authors:** Pengfei Li, Haitang Wu, Zhixia Zhao, Ping Du, Haitong Xu, Hongchuan Liu, Yu Zhou, Weiyue Yu, Hao Li, Lihong Liu

**Affiliations:** ^1^ Pharmacy Department of Beijing Chao-yang Hospital, Capital Medical University, Beijing, China; ^2^ Shanghai Xihua Scientific Co., Ltd, Shanghai, China

**Keywords:** clevidipine, H152/81, LC-MS/MS, whole blood, bioequivalence

## Abstract

Clevidipine is an ultrashort-acting dihydropyridine calcium antagonist, which can control blood pressure accurately. It is necessary to develop a liquid chromatography–tandem mass spectrometry (LC-MS/MS) method to quantitate clevidipine and its active metabolite H152/81 for clinical pharmacokinetic study and therapeutic drug monitoring. Liquid–liquid extraction was used for sample preparation, and clevidipine-d_7_ and H152/81-^13^C-d_3_ were chosen as the isotope internal standard. The chromatographic separation was performed on an ACE Excel 2 Phenyl column (50 × 2.1 mm). Mass quantification was carried out on the multiple reaction monitoring of the transitions of m/z 473.1→338.1, 480.1→338.1, 356.0→324.0, and 362.2→326.2 for clevidipine, clevidipine-d_7_, H152/81, and H152/81-^13^C-d_3_. The validated method gave an excellent linearity over a concentration range of 0.1–30 ng/ml for clevidipine and 2–600 ng/ml for H152/81. Other fully validated content such as accuracy, precision, extraction recovery, matrix effect, and stability were also investigated and showed satisfactory results. It was strongly recommended that whole blood is the first choice for clinical bioanalysis. Using whole blood for sample analysis can reduce the whole blood collection volume (1 ml vs. 4 ml) and shorten the time from sample collection to storage to 5 min, and there is no centrifugation process and precooling in the ice water bath, which can further reduce the instability caused by exposure. The method was successfully applied to a bioequivalence study of clevidipine butyrate-injectable emulsion.

## Introduction

Nowadays, various factors such as smoking, medications, non-compliance, and poorly controlled chronic hypertension result in an acute rise in blood pressure, which leads to multiple damage and complications, such as heart failure, stroke, myocardial infarction, and renal dysfunction (Aggarwal and Khan, 2006; Sarafidis et al., 2012; Samadian et al., 2016; Watson et al., 2018). Acute severe hypertension, different from chronic hypertension in mechanism and treatment responsiveness, requires a more rapid intravenous therapy to control blood pressure effectively, while the use of oral agents is not desirable (Aronson, 2009; Zhou et al., 2014a).

Clevidipine, in the category of arterial vasodilators, is an ultrashort-acting dihydropyridine L-type calcium channel antagonist (Nguyen et al., 2010). The reason why clevidipine is an ideal agent for the rapid control of blood pressure is because it has a rapid onset (2–4 min) and offset (5–15 min) and low incidence of toxicity and does not cause reflex tachycardia (Ahuja and Charap, 2010; Awad and Goldberg, 2010; Keating, 2014). Clevidipine is rapidly degraded and inactivated by esterases in the blood and extravascular tissues to its active metabolite H152/81 (Smith et al., 2012; Wang et al., 2018). A study has indicated that clevidipine and H152/81 caused CYP3A4 induction in hepatocytes; therefore, it was necessary for simultaneous quantitation of clevidipine and its active metabolite H152/81 in clinical trials (Potential and Clevidipine, 2006).

In earlier years, the quantitation of clevidipine and H152/81 has been reported by HPLC with UV or fluorescence detection methods and capillary gas chromatography–mass spectrometry (Ericsson H et al., 1999; Fakt C and Stenhoff H, 1999; Ericsson H et al., 2000). However, owing to time-consumption in the biological sample process and lower sensitivity, these aforementioned methods are not satisfactory for high-throughput detection in pharmacokinetic studies. Nowadays, LC-MS/MS, a novel and popular technology with high selectivity and sensitivity, is widely used for the determination of multiple drugs.

Yujia Zhang et al. developed and fully validated a simultaneous determination method of clevidipine and H152/81 in human plasma, and the method exhibited good linearity over the concentration ranges of 0.100–40.0 ng/ml for clevidipine and 5.00–400 ng/ml for H152/81with NaF and ascorbic acid used as esterase inhibitor and antioxidant, respectively (Zhang Y et al., 2020).

In this study, a rapid, simple, and more reliable LC-MS/MS method was established and validated for simultaneous determination of clevidipine and H152/81 in human whole blood. Based on the result, there was a conclusion that the method provided higher specificity, sensitivity, and short analysis time. The method using whole blood for sample analysis can reduce the whole blood volume and shorten the time from collection to storage to 5 min. The proposed method was successfully applied to a bioequivalence study of 8 mg/h (16 ml/h) clevidipine butyrate-injectable emulsion by intravenous administration, which lasted 30 min in healthy Chinese volunteers under fed conditions.

## Experiment

### Chemicals and Reagents

**FIGURE 1:**
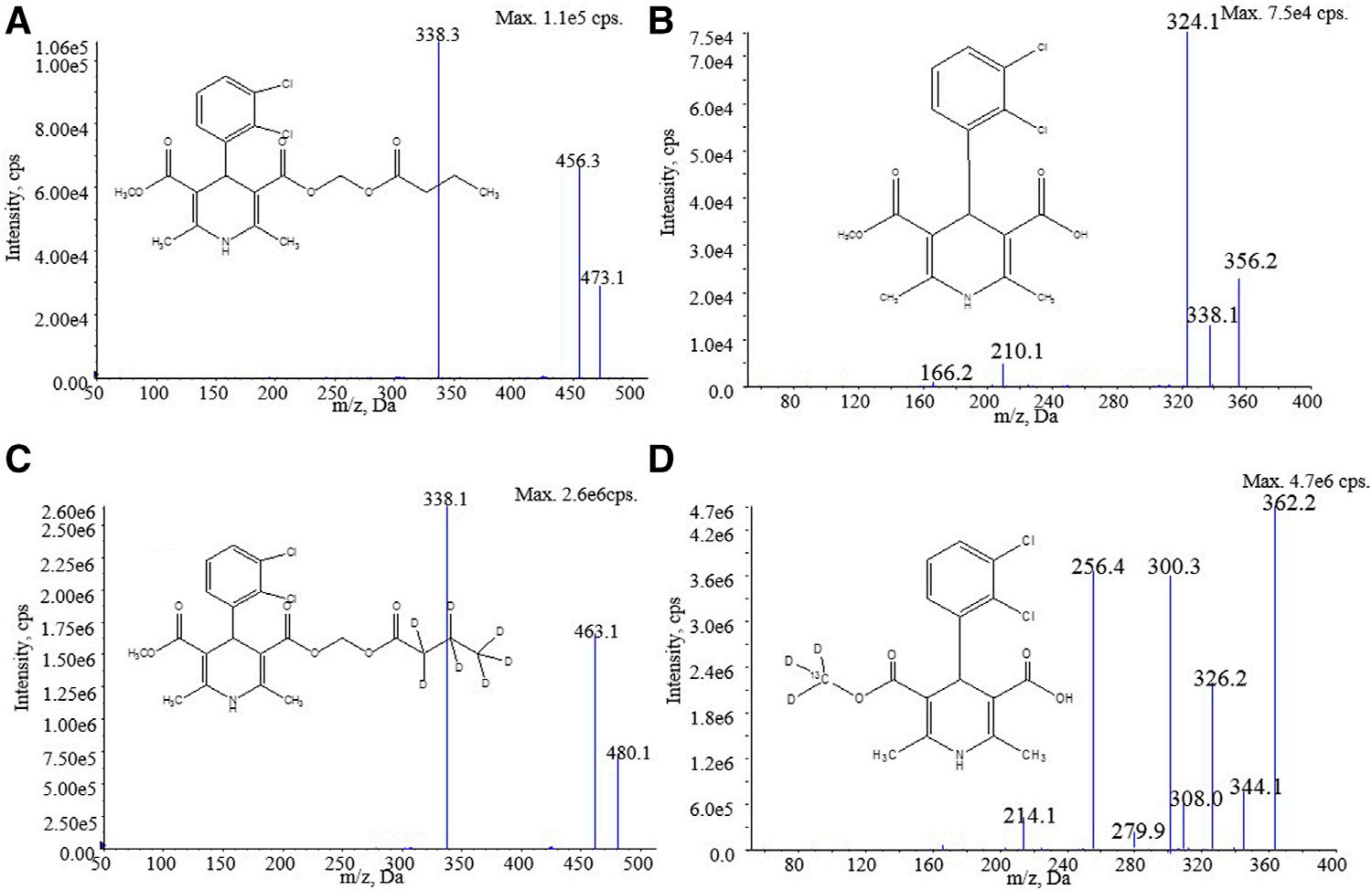
Structure and product ion mass spectra of **(A)** clevidipine, **(B)** H152/81, **(C)** clevidipine-d_7_, and **(D)** H152/81-^13^C-d_3_.

The reference standard of clevidipine (Figure 1, 99.8% purity) and internal standards clevidipine-d_7_ (Figure 1, 99.7% chemical purity and 99.0% isotope purity) and H152/81-^13^C-d_3_ (Figure 1, 99.2% chemical purity and 99.0% isotope purity) were obtained from TLC (TLC Pharmaceutical Standards, Canada). H152/81 (Figure 1, 4-(2, 3-dichlorophenyl)-5-(methoxycarbonyl)-2, 6-dimethyl-1, 4-dihydropyridine-3-carboxylic acid) with 95% purity, was supplied by Shanghai Zzbio Co. Ltd (Shanghai, China). HPLC-grade acetonitrile (ACN) and headspace dimethyl sulfoxide (DMSO) were purchased from Merck (Darmstadt, Germany). MS grade water, formic acid, acetic acid, and HPLC-grade methyl tert-butyl ether (MTBE) were provided by Thermo Fisher Scientific (Fair Lawn, NJ, United States). AR-grade ammonium acetate and ascorbic acid (ACS reagent ≥99%) were procured from Sigma Aldrich Corporation (St. Louis, Missouri). Sodium dodecyl sulfate (SDS) (reagent grade, ≥98%) was purchased from VETEC (Sigma Aldrich Corporation). Human blood was obtained from Chinese volunteers.

### Preparation of Stabilized Human Whole Blood Samples

Ten percent(w/v) SDS solution containing 0.05 M ascorbic acid was added to fresh K_2_EDTA-anticoagulated blood from humans at a ratio of 1:1. After the blood was mixed with the aforementioned stabilizer, the resulting blood (called stabilized blood thenceforth) was finally used for the preparation of calibration standard samples and quality control (QC) samples.

### Preparation of Calibration Standards and Quality Control Samples

Stock solutions (1 mg/ml) of analytes and internal standards were generated by dissolving in DMSO. The standard working solutions of analytes were prepared by serially diluting stock solutions of acetonitrile–water (1:1, v/v). In a similar process, the QC working solutions were prepared with an independent stock solution of analytes. The IS working solutions were prepared by diluting stock solutions of acetonitrile–water (1:1, v/v) at concentrations of 20/200 ng/ml for clevidipine–d_7_/H152/81-^13^C-d_3_. All solutions were stored at −20°C.

Calibration standards and QC samples were prepared by mixing working solutions with stabilized blood to obtain calibration standards, and concentrations of the calibration standards were 0.1/2, 0.2/4, 0.5/10, 2/40, 5/100, 15/300, 27/540, and 30/600 ng/ml for clevidipine/H152/81, and the QC samples (LLOQ QC, low QC, GMQC, medium QC, and high QC) were 0.1/2, 0.3/6, 1.5/30, 8/160, and 24/480 ng/ml for clevidipine/H152/81.

### Sample Preparation

Liquid–liquid extraction (LLE) was used to extract clevidipine and H152/81 from human blood. Aliquots of 25 μl IS working solution, 50 μl blood sample, and 50 μl of 0.1% formic acid were added to 1.5-ml polypropylene tubes precooled in an ice bath. After vortex-mixing for 1 min, 500 μl of MTBE was added to each sample and vigorously vortexed for ten min. Subsequently, the samples were centrifuged at 10,000 g for five min at 4°C. Then, 200 μl of the supernatants were transferred into a clean 96-well plate and evaporated under nitrogen at room temperature. Finally, the residues of the sample were reconstituted in 200 μl of methanol–water (4:6, v/v) containing 2 mM NH_4_AC and 0.025% acetic acid and injected into the LC-MS system.

### LC-MS/MS Conditions

The LC-MS/MS system consists of a Shimadzu UPLC system (two binary pumps LC-30AD, a vacuum degasser DGU-20As, a temperature-controlled column compartment CTO-30A, an autosampler SIL-30AC, and a system controller CBM-20A) connected with an AB SCIEX Triple Quad 6500^+^ mass spectrometer (MS) from Sciex (Framingham, United States). Chromatographic separation was performed on an ACE Excel 2 Phenyl column (50 × 2.1 mm id) maintained at 40°C with 2 mM ammonium acetate in water (mobile phase A) and acetonitrile (mobile phase B) at a flow rate of 0.6 ml/min and injection volume of 20 μl. The gradients were as follows: 0–2.00 min, 30–35% B; 2.00–4.00 min, 35–65% B; 4.00–4.01 min, 65–95% B; 4.01–4.70 min, 95% B; 4.70–4.71 min, 95–30% B; and 4.71–5.50 min, 30% B. Mass quantification was operated in the positive electrospray ionization (ESI) mode. By using the multiple reaction monitoring (MRM) mode, the optimized transitions were m/z 473.1→338.1 for clevidipine, m/z 480.1→338.1 for clevidipine-d_7_, m/z 356.0→324.0 for H152/81, and m/z 362.2→326.2 for H152/81-^13^C-d_3_. The optimal parameters are shown in Table 1.

**TABLE 1:** Optimized parameters for determination of clevidipine and H152/81 using LC-MS/MS.

Parameter	Value	
	Clevidipine clevidipine-d_7_	H152/81 H152/81-^13^C-d_3_
Curtain gas (N_2_, psi)	40	40
CAD gas (unit)	8	8
Ionspray voltage (V)	5,500	5,500
Nebulizer gas (N_2_, psi)	55	55
Heater gas (N_2_, psi)	60	60
Turboheater temperature (°C)	300	300
Declustering potential (V)	35	40
Collision energy (eV)	14	14
EP (V)	10	10
CXP (V)	17	15

### Method Validation

The assay method validation was performed according to the bioanalytical method validation guidelines (U.S. Department of Health and Human Services, FDA, et al., 2018). A full validation (calculation curve, selectivity, accuracy, precision, matrix effect, recovery, dilution integrity, carryover, and stability) was investigated.

The calculation curves contained eight different concentrations at two repeats and were prepared in the biological samples by spiking human whole blood with standard solutions and validated in at least three batches. The range of calculation curves was 0.1–30 ng/ml for clevidipine and 2.0–600 ng/ml for H152/81. The calculation curves were produced by plotting the concentration of the standards against the analyte to IS peak area ratios using least square linear regressions with a weighting factor of 1/x^2^. The acceptance criteria were that 75% (at least six) non-negative calibrators should be with a relative deviation (RE) within ±15% at each concentration (within ± 20% at LLOQ) and the calculation curves involved a coefficient of determination (r^2^) ≥ 0.99.

The selectivity of the bioanalytical method included the interference between human blank whole blood and analytes. Six individual human blank whole blood samples with and without analytes and ISs were compared to investigate the potential interfering peaks at the retention time. The responses of analytes in the blank should not exceed 20% of LLOQ and 5% of the average IS responses of the calibrators and QCs, respectively.

Five concentration levels of QC samples (0.1/2, 0.3/6, 1.5/30, 8/160, and 24/480 ng/ml for clevidipine/H152/81) with six duplicates repeated in three independent batches were used to verify the accuracy and precision of intra- and inter-batch of the method. The accuracy was expressed as the RE with nominal concentration, and the acceptance criterion was that the RE should be within ±15% of the target concentration (within ± 20% at LLOQ). Precision was demonstrated as the relative standard deviation (RSD), which should be ≤15% at each level (≤20% at LLOQ).

To investigate the recovery of analytes and ISs, six replicates from spiked blood samples at three concentration levels (LQC, MQC, and HQC) were used to assess the extraction process, by calculating the mean peak areas required from QC samples to that of the post-extraction of blank spiked blood QC solutions. The matrix effect of the analytes and ISs was evaluated by comparing the responses of the post-extraction six different blank human spiked blood samples with those of the neat solutions at three QC concentration levels (LQC, MQC, and HQC).

Dilution integrity was evaluated by DQC samples (75.0 ng/ml and 1,500 ng/ml for clevidipine and H152/81, respectively) with fivefold dilution to determinate the influence of the accuracy and precision of the dilution procedure. The mean accuracy of the dilution QCs should be within ±15% of the nominal concentration, and the precision should not exceed 15%.

Carryover was investigated by injecting an extracted blank matrix immediately after the upper limit of quantitation (ULOQ) sample. The acceptance criteria were that the peak area at the retention time of analyte in the blank sample should be <20% of the analyte peak area in the LLOQ sample and <5% of the average peak area of IS.

The stability of analytes in the matrix was evaluated by the spiked QC samples (LQC and HQC) with six replicates under the following conditions: room temperature with an ice bath for one h, four freeze–thaw cycles at −80°C, long term at −20°C and −80°C for 6 and 34 days, and the autosampler stability at a setup temperature (8°C) for 146 h. All the samples were analyzed with the freshly prepared calibration curves. The stability of stock and working solutions of analytes and ISs was calculated by comparing the peak area of the stock and working solution storage at −20°C for 35 days with that of freshly prepared solutions.

### Bioequivalence Study and Incurred Sample Reanalysis

The validated method was successfully applied to detect the whole blood concentration of clevidipine and H152/81 after an intravenous administration of 8 mg/h (16 ml/h) which lasted 30 min in 24 healthy Chinese volunteers in the age from 20 to 45 years and a body mass index (BMI) from 19.1 to 25.4 kg/m^2^. The design of the study was a single-center, open-label, randomized, two-period, two-treatment, and a single-dose, crossover bioequivalence study for a test (50 ml: 25 mg clevidipine butyrate-injectable emulsion from Beijing Tide Pharmaceutical Co., Ltd) and a reference (Cleviprex^®^, 50 ml: 25 mg clevidipine butyrate injectable-emulsion from Chiesi United States, Inc.) formulation under fed conditions with a cleaning period of seven days between the two formulations. The protocol and informed consent form were approved by the Ethics Committee of Beijing Chao-yang Hospital, Capital Medical University (BCYH-CMU), with the ethical approval number 2017-drug-8, and all volunteers provided with informed written consent in BCYH-CMU. The whole blood samples (1 ml each) were collected from the forearm vein within 30 min before administration, and 10, 20, 30, 32, 34, 36, 38, 42, 50 min, 1, 1.5, 2.5, 4.5, 8.5, 12.5, 24.5, and 48.5 h after administration, and were then put into a K_2_EDTA blood collection tube, which was preloaded with 1 ml stabilizer ((10% w/v) SDS aqueous solution containing 0.05 M ascorbic acid). The samples were placed slightly upside down 8–10 times directly after collection and stored below −60°C (the refrigerator temperature was set at −70 ± 10°C) until analysis. The pharmacokinetic parameters of clevidipine and H152/81 were evaluated by non-compartmental analysis using Phoenix™ WinNonlin^®^ software version 8.0, and other data were summarized and analyzed using SAS version 9.4 software.

Incurred sample reanalysis (ISR) was performed to verify the reproducibility of the method. In this study, ISR samples include 85 samples for clevidipine and 111 samples for H152/81, of which concentrations were near C_max_ and the elimination phase.

## Results and Discussion

### Method Development

In the mass conditions, the MRM in a positive mode with an ESI source was used to obtain higher sensitivity and better specificity. In the Q1 full scan of the analytes and ISs, the highest intensity signal [M + NH4]^+^ ions at m/z 473.1, 480.1, and [M + H]^+^ ions at m/z 356.0, 362.2 were chosen as the precursor ions for clevidipine, clevidipine-d_7_, H152/81, and H152/81-^13^C-d_3_, respectively. Due to the existence of chlorine, the precursor ion of H152/81 had a serious isotopic interference to the precursor ion at m/z 360 of H152/81-^13^C-d_3_. Selecting the isotope precursor ion at m/z 362.2 of H152/81-^13^C-d_3_ can avoid the isotope interference. The product ion mass spectra of analytes and ISs were optimized under collision-induced dissociation, and the most stable fragment ions were selected at m/z 338.1, 338.1, 324.0, and 326.2 for clevidipine, clevidipine-d_7_, H152/81, and H152/81-^13^C-d_3_, respectively (Figure 1). In addition, the other mass spectrum parameters were also optimized to implement a higher mass response (Table 1).

To obtain the optimal separation of analytes and interferents, appropriation retention time, a better peak shape, and the chromatographic conditions were optimized. The chromatographic column plays a key role in the liquid chromatographic conditions. After a comparison of several different columns, an ACE Excel 2 Phenyl column (50 × 2.1 mm id) which achieved excellent resolution and satisfactory sensitivity was finally selected. Some solvents such as methanol, acetonitrile, water, formic acid, and ammonium acetate were considered for the mobile phase. In the aspect of the elution effect, acetonitrile was much stronger than methanol. Meanwhile, using acetonitrile as the mobile phase could yield lower background noise. The addition of 2 mM ammonium acetate in water could enhance the peak shape of analytes and ISs. Finally, acetonitrile and water containing 2 mM ammonium acetate as mobile phases with a gradient elution at 0.6 ml/min of flow rate provided symmetric peaks, optimal retention time, and higher sensitivity to the analytes and ISs.

As we know, the stable isotope-labeled standard is the best choice as the internal standard due to the similarities in mass and chromatographic behaviors such as retention action, ionization, and extraction efficiency with the analytes. Therefore, using a stable isotope-labeled standard was suitable for improving both the precision and the trueness of methodology in the LC-MS/MS analysis. Thus, clevidipine-d_7_ and H152/81-^13^C-d_3_ were chosen as the internal standards in this study.

Due to the wide presence of esterase, which is located in the membrane or the cytosol of the red blood cells, caused the instability and fast degradation of clevidipine and its metabolites throughout the sample collection, processing, and before sample extraction. It is recommended that the concentration of analytes in whole blood, rather than plasma, is more accurate in quantification (Cao P et al., 2015; Wang Y et al., 2018). Compared with previous reports (Zhang Y et al., 2020), using whole blood for sample analysis can reduce the whole blood collection volume (1 ml vs. 4 ml) and shorten the time from sample collection to storage to 5 min, and there is no centrifugation process and precooling in the ice water bath, which can further reduce the instability caused by exposure. Therefore, we strongly recommend that the whole blood is the first choice for clinical bioanalysis.

As we all know, clevidipine is unstable and can be rapidly hydrolyzed by esterase in whole blood due to its ester group. SDS, a common esterase inhibitor, was demonstrated to prevent clevidipine from hydrolysis distinctly in whole blood, particularly at low temperature (Zhou et al., 2014b; Cao P et al., 2015; Sacks D et al., 2018). In addition, utilizing ascorbic acid was beneficial to protect oxidation. Moreover, after the full validation of the methodology, adding SDS and ascorbic acid had no significant effect on the MS signal of the analyte and IS, and the LLOQ could be as low as 0.1/2 ng/ml for clevidipine/H152/81. Therefore, adding 10% (w/v) SDS and 0.05 M ascorbic acid into human whole blood was performed to minimize enzymatic degradation and oxidation of clevidipine and H152/81 in this study, and subsequently, the method was applied to the clinical trial successfully.

Compared with protein precipitation, the LLE method displayed fewer interference components, better signal-to-noise ratio, and a higher extraction recovery for sample preparation. Regular different extraction solvents and different numbers of pH modifiers were assessed to improve the LLE procedure in this study. Compared with ethyl acetate, the recovery of MTBE remained at the same high level, and the matrix interference caused by emulsification was also significantly reduced. Eventually, MTBE was chosen as the optimum LLE agent and 0.1% FA was utilized to obtain high and stable extraction efficiency.

### Method Validation

The calculation curve contained eight different concentrations and showed excellent linearity in the concentration range of 0.1–30 ng/ml for clevidipine and 2.0–600 ng/ml for H152/81. The correlation coefficients of clevidipine and H152/81 were better than 0.9951 in all the validation batches. The LLOQ of clevidipine and H152/81 was 0.1 ng/ml and 2.0 ng/ml (signal-to-noise ratio >10) respectively, which were sufficient for the quantification of clevidipine and H152/81 in the human whole blood sample study.

**FIGURE 2:**
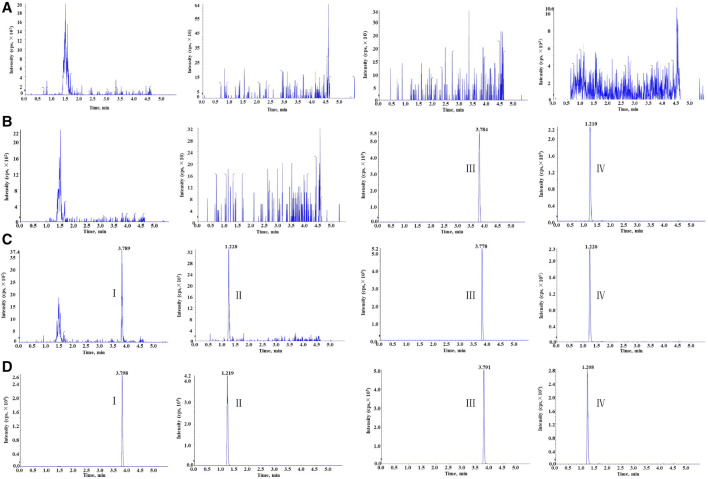
Representative chromatogram: **(A)** blank human blood, **(B)** blank human blood with working solution of ISs, **(C)** blank human blood spiked at the LLOQ, and **(D)** real subject sample at C_max_ concentration for clevidipine (I), H152/81 (II), clevidipine-d_7_ (III), and H152/81-^13^C-d_3_ (IV).

The selectivity of this method was evaluated by comparing the human blank whole blood samples collected from six different sources with the corresponding spiked samples. As Figure 2 shows, the retention time of clevidipine, H152/81, clevidipine-d_7_, and H152/81-^13^C-d_3_ was 3.79, 1.20, 3.78, and 1.19 min, respectively. There was no distinct interference at the retention times of analytes and ISs in the human blank whole blood, which indicated that the method displayed superior selectivity.

The intra- and inter-batch accuracies were within the range of -2.5 to 4.7% and the intra- and inter-batch precisions were less than 6.1% and 7.3% with six duplicates repeated in three independent batches (Table 2). These results conformed to the acceptance criteria toward precision and accuracy, which demonstrated that the precision and accuracy were fit enough for the determination of clevidipine and H152/81 in human blood.

**TABLE 2:** Intra- and inter-batch precision and accuracy of analytes in human blood.

Analyte	Spiked con. (Ng/mL)	Precision (RSD %)			Accuracy (RE %)	
		Intra-batch (n = 6)	Inter-batch (n = 18)		Intra-batch (n = 6)	Inter-batch (n = 18)
Clevidipine	0.1	3.3	5.8		−0.4	0.0
	0.3	4.7	3.5		2.0	2.3
	1.5	2.4	3.7		2.7	4.7
	8.0	3.2	3.0		0.0	1.1
	24	2.1	3.5		0.0	0.8
H152/81	2.0	6.1	7.3		−0.4	−2.5
	6.0	2.5	3.3		0.5	0.5
	30	2.0	3.7		2.0	4.0
	160	5.5	4.4		0.0	0.6
	480	4.4	3.3		1.3	1.7

RSD: relative standard deviation; RE: relative deviation.

The extraction recoveries in low-, medium-, and high-QC samples were 80.3, 83.4, and 80.4% for clevidipine and 76.8, 78.6, and 80.6% for H152/81, respectively, while the extraction recoveries of ISs were 82.5 and 79.0% for clevidipine-d_7_ and H152/81-^13^C-d_3_, respectively. The matrix effects for clevidipine were 114, 117, and 115% at the concentrations of 0.3, 8.0, and 24 ng/ml, respectively, and those for H152/81 were 98.8, 97.8, and 101% at the concentrations of 6, 160, and 480 ng/ml, respectively. These results indicated that no matrix effect for both analytes and ISs were seen in the method in which the recovery was consistent and reproducible.

The accuracy of the DQC samples ranged from 101.5 to 105.6% for clevidipine and 96.7 to 105.3% for H152/81, and the precision of clevidipine and H152/81 was 1.5% and 2.9%, respectively, indicating that there was no significant effect on dilution to quantify analytes before sample analysis.

The peak area at the retention time of the analytes and ISs in matrix blank samples injected after the ULOQ samples had no obvious interference, which was within ±20% of the analyte peak area in the LLOQ samples and within ±5% of the average peak area of ISs.

The stock and working solutions of the analytes were stable with storage at −20°C for 34 days. The results of the stability of clevidipine and H152/81 in human blood (Table 3) showed that no visible degradation occurred under either of the examined conditions, which showed that there was no stability issue for the detection of the analytes in this study.

**TABLE 3:** Stability of analytes under examined conditions (n = 6).

Analyte	Concentration (ng/ml)	Stability (RE %)							
		Room temperature		Freeze–thaw		Long-term			In autosampler
		Ice bath for 1 h		Four cycles		−20°C (6 days)	—80°C (34 days)		8°C for 146 h
Clevidipine	0.3	3.3		2.6		4.2	4.8		3.9
	24	3.2		0.9		1.8	1.2		3.5
H152/81	6.0	5.3		4.2		10.8	6.1		6.6
	480	5.1		3.9		2.9	2.6		3.6

RE: relative deviation.

### Application to a Bioequivalence Study

**FIGURE 3:**
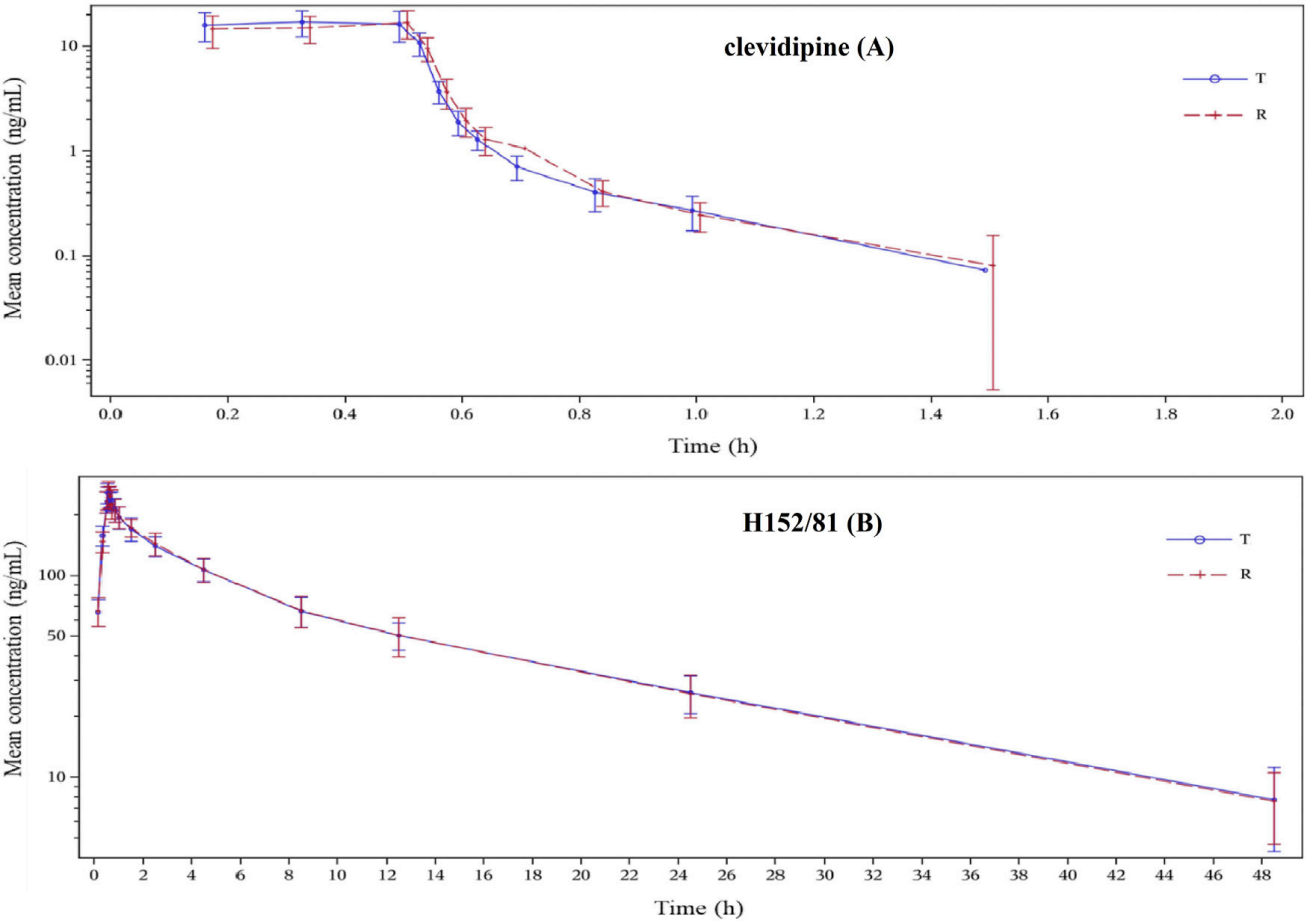
Blood concentration–time semi-logarithmic curves of the test and reference formulations of clevidipine **(A)** and H152/81 **(B)** in the fed state in healthy Chinese volunteers.

In this study, because of the occurrence of adverse events, 23 of the 24 subjects in the reference group and 21 of the 24 subjects in the test group were eventually incorporated into the pharmacokinetic analysis, while 20 of the 24 subjects were finally brought into the bioequivalence study. The reasons for the abscission of three subjects were the changes of the ST-T segment of the ECG before the second administration, the decrease in plasma fibrinogen before the cleaning period, the increase in the heart rate and blood pressure, which appeared six min after the first administration. The average blood concentration–time semi-logarithmic curves of clevidipine and H152/81 obtained for test and reference formulations are displayed in Figure 3, respectively. The mean pharmacokinetic parameters of clevidipine and H152/81 with both formulations are generalized in Table 4, respectively. According to the result, the ratios of the geometric mean log-transformed parameters, C_max_, AUC_0−t_, AUC_0−∞_, and their 90% confidence intervals ranged from 97.57 to 119.86% for clevidipine and 97.62 to 106.46% for H152/81, which were within the acceptance criterion of 80–125%, showing the bioequivalence of the test formulation with the reference product in terms of rate and extent of absorption. Meanwhile, there was no significant difference appeared in the variance analysis between the period and sequence.

**TABLE 4:** Main pharmacokinetics parameters of clevidipine and H152/81 after being intravenously administered 8 mg/h test or reference clevidipine butyrate-injectable emulsion lasted 30 min.

Analyte	Parameter	Mean±SD		Ratio (T/R, %) (n = 20)	90% CI (lower-upper) (n = 20)	Intra-subject variation (%) (n = 20)	Power (%) (n = 20)
		Test (N = 21)	References (N = 23)				
Clevidipine	C_max_ (ng/ml)	17.74 ± 4.167	17.723 ± 8.0233	109.56	100.33–119.63	16.1	82.1
	AUC_0-t_ (h·ng/mL)	7.5103 ± 1.8597	7.2518 ± 2.2651	108.14	97.57–119.86	18.8	77.8
	AUC_0-∞_ (h·ng/mL)	7.5795 ± 1.8707	7.3184 ± 2.2726	108.09	97.60–119.71	18.7	78.6
	^a^T_max_ (h)	0.3333 [0.1661, 0.5336]	0.3333 [0.1544, 0.5006]	—	—	—	—
	λ_z_ (1/h)	2.8696 ± 1.3007	2.7790 ± 1.3802	—	—	—	—
	t_1/2_ (h)	0.2945 ± 0.1362	0.3102 ± 0.1361	—	—	—	—
H152/81	C_max_ (ng/ml)	264.3 ± 32.03	262.7 ± 37.71	100.91	97.62–104.31	6.0	100.0
	AUC_0-t_ (h·ng/mL)	2,141.5127 ± 254.1394	2,118.5778 ± 301.2615	102.00	98.09–106.07	7.1	100.0
	AUC_0-∞_ (h·ng/mL)	2,287.7115 ± 320.7648	2,257.1306 ± 351.1483	102.40	98.50–106.46	7.1	100.0
	^a^T_max_ (h)	0.5664 [0.5003, 0.7000]	0.5678 [0.5006, 0.7008]	—	—	—	—
	λ_z_ (1/h)	0.0569 ± 0.0108	0.0565 ± 0.0086	—	—	—	—
	t_1/2_ (h)	12.6910 ± 2.8949	12.5664 ± 2.0300	—	—	—	—

a T_max_ was represented by the median [minimum, maximum]. C_max_: peak plasma concentration; AUC: area under the curve; T_max_: time of C_max_; λ_z_: elimination rate constant; t_1/2_: terminal half-life.

The percent change of incurred samples was within ±20% that compared with the same initial samples. The ISR result indicated the perfect reproducibility and reliability of this method.

## Conclusion

To the best of our knowledge, this is the first time that a rapid and sensitive LC-MS/MS method was developed and validated for the simultaneous quantitation of clevidipine and its active metabolite H152/81 in human whole blood for bioequivalence study. It is strongly recommended that whole blood is the first choice for clinical bioanalysis. Collecting whole blood samples greatly reduces the blood sample collection volume and the time from collection to storage, and there is no centrifugation process and precooling in the ice water bath, which can further reduce the instability caused by exposure. The method with the LLE sample preparation process minimized the strong matrix effect caused by endogenous substances and had a lower LLOQ (0.1 ng/ml for clevidipine and 2.0 ng/ml for H152/81). Moreover, using SDS and ascorbic acid can effectively reduce the hydrolysis and oxidation of clevidipine and H152/81 during analysis. Eventually, the method was successfully applied to the bioequivalence study in healthy Chinese volunteers.

## Data Availability

The original contributions presented in the study are included in the article/Supplementary Material, further inquiries can be directed to the corresponding authors.
